# Development of Genome-Wide SNP Markers for Barley via Reference- Based RNA-Seq Analysis

**DOI:** 10.3389/fpls.2019.00577

**Published:** 2019-05-10

**Authors:** Tsuyoshi Tanaka, Goro Ishikawa, Eri Ogiso-Tanaka, Takashi Yanagisawa, Kazuhiro Sato

**Affiliations:** ^1^Breeding Informatics Research Unit, Division of Basic Research, Institute of Crop Science, National Agriculture and Food Research Organization (NARO), Tsukuba, Japan; ^2^Bioinformatics Team, Advanced Analysis Center, National Agriculture and Food Research Organization (NARO), Tsukuba, Japan; ^3^Advanced Agricultural Technology and Sciences, Graduate School of Life and Environmental Sciences, University of Tsukuba, Tsukuba, Japan; ^4^Breeding Strategies Research Unit, Division of Basic Research, Institute of Crop Science, National Agriculture and Food Research Organization (NARO), Tsukuba, Japan; ^5^Soybean and Field Crop Applied Genomics Research Unit, Division of Field Crop Research, Institute of Crop Science, National Agriculture and Food Research Organization (NARO), Tsukuba, Japan; ^6^Wheat and Barley Breeding Unit, Division of Wheat and Barley Research, Institute of Crop Science, National Agriculture and Food Research Organization (NARO), Tsukuba, Japan; ^7^Group of Genome Diversity, Institute of Plant Science and Resources, Okayama University, Okayama, Japan

**Keywords:** barley, genotyping, RNA-Seq, Japanese barley breeding, amplicon sequencing

## Abstract

Marker-assisted selection of crop plants requires DNA markers that can distinguish between the closely related strains often used in breeding. The availability of reference genome sequence facilitates the generation of markers, by elucidating the genomic positions of new markers as well as of their neighboring sequences. In 2017, a high quality genome sequence was released for the six-row barley (*Hordeum vulgare*) cultivar Morex. Here, we developed a *de novo* RNA-Seq-based genotyping procedure for barley strains used in Japanese breeding programs. Using RNA samples from the seedling shoot, seedling root, and immature flower spike, we mapped next-generation sequencing reads onto the transcribed regions, which correspond to ∼590 Mb of the whole ∼4.8-Gbp reference genome sequence. Using 150 samples from 108 strains, we detected 181,567 SNPs and 45,135 indels located in the 28,939 transcribed regions distributed throughout the Morex genome. We evaluated the quality of this polymorphism detection approach by analyzing 387 RNA-Seq-derived SNPs using amplicon sequencing. More than 85% of the RNA-Seq SNPs were validated using the highly redundant reads from the amplicon sequencing, although half of the indels and multiple-allele loci showed different polymorphisms between the platforms. These results demonstrated that our RNA-Seq-based *de novo* polymorphism detection system generates genome-wide markers, even in the closely related barley genotypes used in breeding programs.

## Introduction

The release of the draft barley (*Hordeum vulgare*) genome (International Barley Genome Sequencing Consortium [IBSC], 2012) revealed the existence of a large number of sequence polymorphisms (∼15 million) between several major haplotypes of this crop, even within exonic regions (∼350,000). The identification of these candidate marker polymorphisms encouraged us to generate a whole-genome genotyping system for barley. The barley research community has developed a number of genome marker-based systems, initially using sequences from expressed sequence tags (ESTs) generated by the international consortium using regional donor cultivars of barley. The first comprehensive polymorphism detection system was Affymetrix GeneChip Barley1 (Close et al., 2004; Luo et al., 2007; Chen et al., 2010; Moscou et al., 2011), which uses hybridization probe sequences chosen to avoid the polymorphic regions of transcripts, and enables the simultaneous detection of gene expression and polymorphisms. Transcript sequence polymorphisms were used to develop the Illumina GoldenGate Assay SNP detection system, which included 2,943 mapped SNPs (Close et al., 2009), and other high-density marker systems were also created for the Illumina iSelect platform (Bayer et al., 2017). These prefixed marker systems contributed to the identification of genome-wide consensus marker polymorphisms by the barley research community, and also promoted the sequencing of the barley genome by facilitating the genetic mapping of BAC clones onto the genome (International Barley Genome Sequencing Consortium [IBSC], 2012). However, using sequence polymorphisms derived from EST donors limited the application of these marker systems, particularly in terms of marker detection using alien sources of materials.

Marker-assisted selection has become an important technique in crop breeding. Marker systems have been successfully applied to the selection of traits in a population generated from crosses between distantly related parents; however, relatively few markers are available for distinguishing between closely related strains, especially between the highly advanced parents used in breeding (Sato et al., 2011). The poor detection of markers in these populations is mainly due to the ascertainment bias in the source of the polymorphisms (Moragues et al., 2010).

The least biased method for detecting polymorphisms is to sequence haplotypes and compare their sequences. Next-generation sequencing (NGS) platforms have been used to resequence the haplotypes of many families (Wang et al., 2018; Zhao et al., 2018); however, sequencing the entire genome of barley is more difficult to assemble and analyze the sequences due to its large and repetitive genome. Even without reference genome sequences, NGS can provide sequence-based genome-wide genotyping data sets. For the partial sequencing of a genome, restriction site-associated DNA sequencing (RAD-Seq) and genotyping-by-sequencing (GBS) technologies utilize restriction enzymes to identify high-density polymorphisms in the sequences around the digested regions (Poland et al., 2012; Kobayashi et al., 2016).

RNA-Seq was initially developed to analyze the expression levels of genes, but is also used for the detection of SNPs in the transcribed regions of the barley genome (Haseneyer et al., 2011; Takahagi et al., 2016). RNA-seq is a potential strategy to genotype species with a large genome size, where direct resequencing is too expensive. Previous work in wild wheat (Nishijima et al., 2016) and human (Piskol et al., 2013) demonstrated the utility of RNA-seq as a robust method to identify polymorphisms in large genome size samples. RNA sequences are only derived from exon sequences; therefore, they can be used to generate markers specific to genic regions, which are more likely to cause a phenotypic change that can be exploited or avoided in crop breeding. The total number of expressed genes is estimated to be ∼30,000, with an average transcript size of ∼1.5 Kb, providing a rough estimate of a single coverage of approximately 45 Mb from the full-length cDNA sequencing projects in barley (Sato et al., 2009; Matsumoto et al., 2011). The cost and time involved in RNA-Seq are much less than those required for whole-genome sequencing, particularly when genotyping multiple haplotypes for the detection of polymorphisms. The number of reads generated using RNA-Seq depends on the expression of each gene in the sequenced organs or in response to the particular growth conditions; therefore, the quality of markers must be confirmed, particularly for genes with a lower gene expression and therefore lower sequence redundancy.

In this report, we developed an RNA-Seq-based genotyping pipeline focusing on the genic sequences of the reference genome. Using this method, we evaluate whether we can reduce the calculation time required for genotyping without reducing the quality and accuracy of the results. We also compare and agree the results of our RNA-Seq-based genotyping with those generated using an alternative platform, amplicon sequencing (AmpliSeq).

## Materials and Methods

### Samples for RNA-Seq

Public Japanese barley breeding (*H. vulgare*) programs provided the major strains used in their programs for genotyping. These breeding programs focused on six-row hulled food barley, hull-less food barley, two-row non-malting barley, and two-row malting barley strains. We constructed one library of RNA-Seq for 68 accessions, two libraries for 38 accessions and three libraries for two accessions. A total of 150 RNA-Seq libraries were used in this study (Supplementary Table 1).

### RNA Extraction, Library Preparation, and Sequencing

The methods for growing the plants, RNA isolation, library preparation, and RNA sequencing were described by Sato et al. (2016). In brief, the seedling shoot and root tissues were sampled from plants with 5-cm shoots. RNA was also isolated from the immature spike within the leaf sheath of 39 strains, 5 days before heading in plants grown in the glasshouse. The RNA-Seq library was sequenced using the MiSeq Reagent Kit V3 (2 × 300 bp cycles) on a MiSeq NGS system according to the MiSeq System User Guide (Illumina, CA, United States), and fastq files with a read length of 300 bp were obtained from both ends of the fragments. The data were registered in the DDBJ BioProject (Accession: PRJDB6775).

### Genotyping Using RNA-Seq Data

**FIGURE 1:**
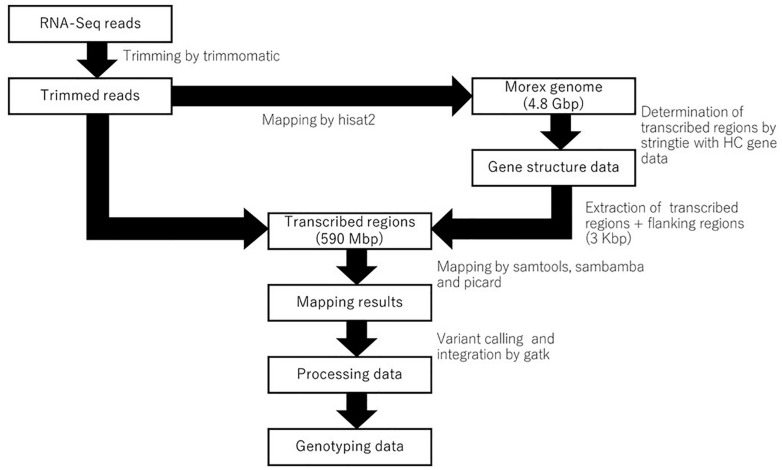
A pipeline of RNA-seq data for genotyping on the transcribed regions of reference genome cv. Morex.

The pipeline for genotyping using RNA-Seq is shown in Figure 1. The reference genome sequence of barley cultivar Morex and the annotation data were obtained from the Plant Genomics and Phenomics Research Data Repository^1^ (Mascher et al., 2017). The raw RNA-Seq data were processed to remove the adapter sequences and low-quality bases using trimmomatic-0.30, with the option “ILLUMINACLIP:adapter.fa:2:30:10 LEADING:15 TRAILING:15 SLIDINGWINDOW:4:15 MINLEN:32” (Bolger et al., 2014). Owing to the difficulty of the indexing large genome sequences onto chromosomes, every chromosome was split into two sections. The trimmed paired reads were then mapped onto the genome using hisat2-2.0.5 with the option “–min-intronlen 20 –max-intronlen 10000 –downstream-transcriptome-assembly –rna-strandness RF” (Kim et al., 2015). The resulting mapping of each library was processed using samtools-1.4 (Li, 2011), sambamba (Tarasov et al., 2015) and picard^2^. Gene models based on known high-confidence (HC) genes (Mascher et al., 2017) were determined from all RNA-Seq samples using stringtie-1.3.3 (Pertea et al., 2015), after which the transcribed regions, including the exons, introns, and 3-Kbp upstream/downstream regions, were extracted from the reference genome sequence (Mascher et al., 2017) and referred to as “transcribed regions.” In addition to mapping the sequence data to the reference genome, the data were also mapped to the transcribed regions using hisat2-2.0.5. Each sample was genotyped using GenomeAnalysisTK-3.2-2 with the option “-T HaplotypeCaller –emitRefConfidence GVCF –variant_index_type LINEAR –variant_index_parameter 128000 –filter_reads_with_N_cigar,” and a gvcf file was constructed. Finally, all genotyping results were merged into a single file using GenomeAnalysisTK-3.2-2 (McKenna et al., 2010). The results were filtered by > 1 read depth and no neighbor polymorphisms around 60 bp using Perl scripts (Supplementary Table 2). When there were two or more RNA-Seq libraries for an accession, we used the seedling shoot and root library which was common to all accessions.

### Library Preparation and Sequencing for AmpliSeq

Of the 108 accessions, 38 strains were randomly selected for AmpliSeq. The genomic DNA of each accession was extracted from ∼100 mg of young leaf tissue using a DNeasy Plant Mini Kit (Qiagen, Hilden, Germany), and was quantified using a Qubit dsDNA High Sensitivity Assay Kit (Thermo Fisher Scientific, Waltham, MA, United States). The library was constructed using the Ion AmpliSeq Library Kit 2.0 (Thermo Fisher Scientific), according to the manufacturer’s protocol. Using a multiplex PCR, 10 ng of each genomic DNA sample was amplified with a custom amplicon panel. Each sample was amplified in a 10-μL solution containing 2 μL 5× Ion AmpliSeq HiFi Master Mix, 5 μL 2× AmpliSeq Custom Primer Pool, 10 ng DNA, and nuclease-free water. The reaction mix was heated for 2 min at 99°C to activate the enzyme, followed by 18 two-step cycles at 99°C for 15 s and at 60°C for 4 min, and ending with a holding period at 10°C. The amplified samples were digested with 1 μL FuPa enzyme at 55°C for 10 min, after which the enzyme was inactivated with a treatment at 60°C for 20 min. To enable multiple libraries to be loaded on a single chip, 1 μL of a unique diluted mix, including IonCode Barcode and Ion P1 Adapters, was ligated to the end of the digested amplicons using 1 μL DNA ligase at 22°C for 30 min, after which the ligase was inactivated by a 10-min treatment at 72°C. The resulting unamplified adapter-ligated libraries were purified using 22.5 μL of Agencourt AMPure XP Reagent (Beckman Coulter, Brea, CA, United States), after which 75 μL freshly prepared 70% ethanol was added to each library. After purification, the libraries were further amplified to enrich the material for accurate quantification using 25 μL Platinum PCR SuperMix High Fidelity and 1 μL Library Amplification Equalizer Primer Mix (Ion AmpliSeq Library Kit 2.0; Thermo Fisher Scientific) at 98°C for 2 min, followed by five two-step cycles at 98°C for 15 s and 60°C for 1 min. The amplified libraries were then equalized to 100 pM using an Ion Library Equalizer Kit (Thermo Fisher Scientific), and subsequently sequenced on an Ion S5 system using an Ion 540 Chip (Thermo Fisher Scientific), following the manufacturer’s instructions.

### Data Analysis Using Ion Torrent Suite Software

The Ion S5 sequence data was mapped to the transcribed regions using the Ion Torrent Suite version 5.8.0 software. The software was optimized for the Ion Torrent raw data analysis: alignment (Torrent Mapping Alignment Program (TMAP) version 5.8.17), coverage analysis version 5.8.0.8, and variant calling using the Torrent Variant Caller (TVC) plug-in version 5.8.0.8. The variant calling was performed using the default germline parameters.

## Results

### Transcribed Region Sequences Showed Good Performance for RNA-Seq Mapping

We obtained 2.7–10.5 million paired reads of RNA-Seq data from each sample (Supplementary Table 1). On average, more than 5 million paired-end reads were used for genotyping, after being trimmed to remove low-quality and adapter sequences. The maximum trimming rate was 5.9% among the samples. The RNA-Seq reads were mapped onto the reference genome sequence of the barley cultivar Morex (Mascher et al., 2017), with a mapping ratio of 79.4 to 93.4% (Supplementary Table 1). After combining the mapping results with known annotated genes (Mascher et al., 2017), the numbers of predicted transcripts in each sample ranged from 41,028 to 78,200. A Spearman’s rank correlation coefficient between the read numbers and the numbers of predicted transcripts among samples was 0.597. The plot of the read numbers and the numbers of predicted transcripts among samples indicated that the numbers of predicted transcripts were saturated at higher read number samples (Supplementary Figure 1). Among these transcripts, 106,912 loci were identified, including 39,270 known HC loci and 67,642 tentative loci determined using RNA-Seq data from this study. This result suggested that the transcribed regions of Morex were not fully covered by the sequences of the reported HC loci. We also found that the RNA-Seq data of this study did not map on 9,034 HC loci. We tried to define the transcribed regions of our RNA-Seq data on the reference genome sequence; however, sequences obtained using RNA-Seq often lack the start/end positions of the transcripts. We therefore used a set of alternative target sequences named transcribed regions, which included the transcripts, introns, and 3-Kbp upstream/downstream sequences. A number of loci were then concatenated, and a total of 590,551,456 bp of transcribed regions in 45,978 genomic loci were ultimately extracted. These sequences covered ∼12% of the Morex reference genome, 2.64 times more than those of the HC loci (223,654,512 bp) (Mascher et al., 2017).

We mapped the RNA-Seq data onto the transcribed regions, and the resulting mapping ratios differed from those on the reference genome (−22.3 to 1.46%) (Supplementary Table 1). We also found that six samples showed reduction of more than a 5% ratio of “properly mapped reads (without multiple hits)” (referred by hisat2 statistics) on the transcribed regions than the reference genome (Supplementary Table 1). In contrast, 120 of the 150 samples had a better mapping ratio for the transcribed regions than the reference genome (0.07 to 3.40%). These differences were mainly caused by reads with multiple mapping positions.

**FIGURE 2:**
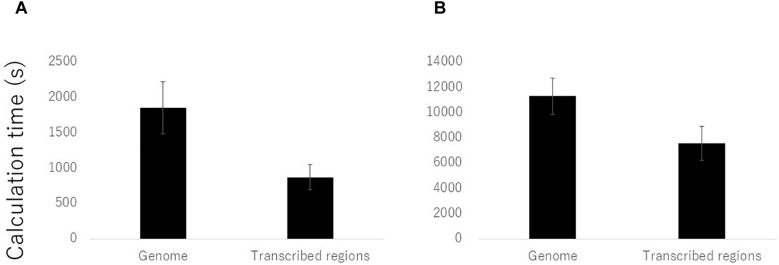
Reduction of calculation time for mapping and genotyping of RNA-Seq data on the transcribed regions compared to the reference genome sequences. Average calculation times of ten randomly selected samples by **(A)** hisat2 and **(B)** gatk software’s were presented.

We compared the calculation times required for mapping and genotyping using the procedures for both the entire reference genome and the transcribed regions. Of the 108 samples, we randomly selected ten samples and calculated the average times required for the hisat2 and gatk analyses. The average time taken when using the transcribed regions was reduced by almost half using hisat2 and by two thirds using gatk in comparison with the times required when using the reference genome (Figure 2). In conclusion, use of only transcribed regions for the genotyping by RNA-Seq was effective in barley by the reduced cost and time compared to the use of reference genome sequence.

### Genome-Wide Polymorphism Detection Among 108 Japanese Barley Strains From RNA-Seq

**FIGURE 3:**
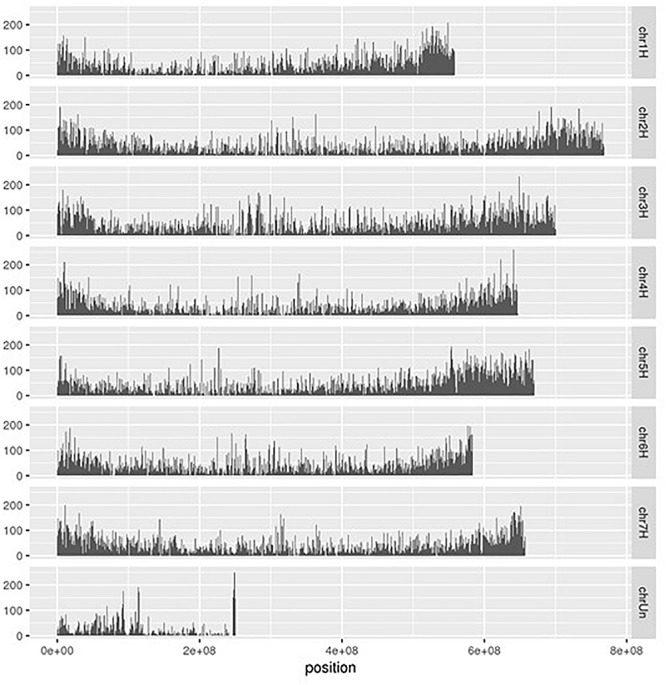
Distribution of polymorphisms detected among RNA-Seq data from 108 Japanese barley strains on the chromosomal positions of reference genome of cv. Morex. The polymorphisms were genotyped by gatk and filtered by the thresholds with > 1 read depth and no neighbor polymorphisms around 60 bp.

Using the RNA-Seq mapping results, we detected 2,214,448 polymorphisms on 42,616 of the 45,978 transcribed regions (92.7%) in the reference genome (Table 1). These polymorphisms were categorized into 1,802,336 SNPs, 354,903 indels, and 57,209 loci with multiple alleles. Of the detected polymorphisms, 493,657 sites were homozygous between the Japanese barley strains and Morex; however, 56.2% of the homozygous polymorphisms were only obtained in a single strain. Of these, 57,209 were loci with two or more alleles. We considered that the polymorphisms with only one heterozygous strain might not be suitable for genotyping, and therefore discarded the polymorphisms only seen in a single strain, heterozygous calls with a single read, and different calls from multiple strains, leaving a total of 1,102,109 SNPs and 200,945 indels remaining. Finally, we extracted 181,567 SNPs and 45,135 indels without sequence polymorphisms and their neighboring 60 bp on both sides. These polymorphisms were located on 28,939 transcribed regions and distributed across the entire reference genome of Morex (Figure 3). The polymorphisms exhibiting differences between the Japanese breeding strains in the pairwise comparison included 44 to 24,026 SNPs and 92 to 2,679 indels (Supplementary Table 3).

**TABLE 1:** Polymorphisms detected between RNA-Seq data from 108 Japanese barley strains and the reference genome sequence of cv. Morex.

Type	Total	No. of SNPs	No. of indels
Detected polymorphisms	2,214,448		
Reliable polymorphisms	1,303,054	1,102,109	200,945
Polymorphisms after processing	226,702	181,567	45,135

We compared the polymorphisms among strains of two-row or six-row barley. In each row type, we filtered out the polymorphisms under the thresholds of both <0.1 minor allele frequency and <0.5 missing genotypes, resulting in a total of 8,475 SNPs and 597 indels remaining. When these polymorphisms were arranged on the chromosomes of Morex (Supplementary Table 4), it was revealed that large regions did not contain any polymorphisms. For example, 981 regions showed more than 1 Mbp without polymorphisms, with a maximum region of 97,779,576 bp to 400,251,595 (302,472,019 bp in length) bp on the sequence of chromosome 4H. These regions might be derived from either the conserved regions within Japanese two-row and six-row barley, or non-transcribed regions.

### Evaluation of the Polymorphisms Detected Using the Two Methods

As described above, the quality of our mapping and genotyping procedures was initially estimated based on the read depth. We further evaluated the quality of the detected polymorphisms using two additional methods. First, we evaluated the sequence polymorphisms derived from multiple RNA samples of a single strain. Of the 108 strains, 38 had two RNA-Seq libraries and reads. If the polymorphisms differed between the libraries, we considered the polymorphisms to be unreliable. We counted the number of agreed (identical) and disagreed (different) polymorphisms between the multiple libraries and calculated their agreement rate (agreed/total polymorphisms), comparing a total of 722,380 to 955,498 polymorphisms for each of the 38 strains. Of these, we omitted around 60% of the polymorphisms because they were detected in only one library. The agreement rates were 84.9 to 95.1% (average 91.1%).

**FIGURE 4:**
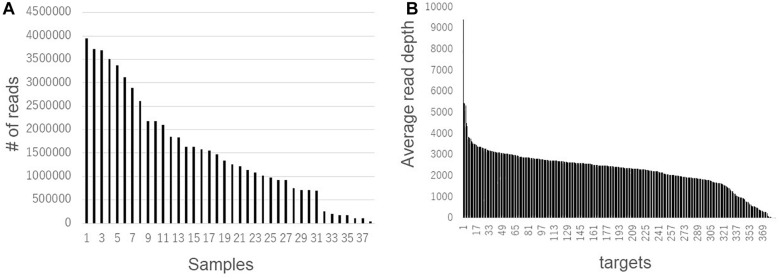
Read number variations among samples and target sites detected by AmpliSeq. Total read number in each sample **(A)** and average read number of 38 samples in each target site **(B)** were shown.

Second, we compared the genotyping data generated using RNA-Seq and AmpliSeq. Although the agreement rate between multiple libraries from a single strain was more than 90% when comparing the RNA-Seq data, systematic genotyping errors could be present in the RNA-Seq polymorphism detection pipeline. To estimate the accurate nucleotide sequence of the polymorphic position, we used AmpliSeq to conduct a highly redundant targeted resequencing of a limited number of polymorphisms derived from the RNA-Seq analysis. Based on 274 randomly selected SNPs and 113 randomly selected indels from the RNA-Seq analysis, we designed 384 primer sets for AmpliSeq. Of these, three primer sets contained multiple (two) polymorphisms. Among the 108 strains, 38 were randomly selected for resequencing. Using two runs of sequencing, a total of 58,693,508 reads were generated and assigned to their respective strains using barcodes. The read number for each strain ranged from 42,702 to 3,942,476 (Figure 4A). The average read depths at a target position were 35 to 366,750, and 373 positions showed more than a × 100 read depth on average (Figure 4B); the 11 positions with less than a × 100 read depth were omitted from the subsequent analysis. The calls at the target positions were compared between the results of the AmpliSeq and RNA-Seq (Table 2). The agreement rates among the strains ranged from 58.2 to 94.6%, and 34 strains showed more than a 90% agreement. The SNPs (93.1%) showed higher agreement rates than the indels (65.1%). Among the above-mentioned 34 strains showing a high level of agreement, the SNP-specific agreement rate was more than 95%. We identified different indel polymorphisms in the RNA-Seq analysis, suggesting the presence of multiple allelic polymorphisms. Several of the SNPs detected using RNA-Seq also contained indels. These results show that AmpliSeq is suitable for the detection of a wider variety of polymorphisms than RNA-Seq, and the number of reads used for AmpliSeq does not affect the overall accuracy of genotyping.

**TABLE 2:** Comparison of the genotyping results between RNA-Seq and AmpliSeq.

Strain	True/false ratio (%)			True		False		Not detected
	Total	SNP	Indel	SNP	Indel	SNP	Indel	
Kashimamugi	94.595	96.465	79.167	191	19	7	5	131
Ishukushirazu	94.492	96.651	77.778	202	21	7	6	117
Misato Golden	94.231	96.774	72.727	180	16	6	6	144
Sayakaze	93.269	96.721	68.000	177	17	6	8	145
Taishomugi	93.133	96.552	70.000	196	21	7	9	119
Fibersnow	93.088	96.216	75.000	178	24	7	8	136
Shunrei	93.074	96.098	69.231	197	18	8	8	119
Ryofu	93.004	96.667	69.697	203	23	7	10	110
Akashinriki	92.991	96.316	66.667	183	16	7	8	139
Sukai Golden	92.829	96.429	62.963	216	17	8	10	102
Harushirane	92.803	95.575	76.316	216	29	10	9	89
Kanto-kawa 98	92.771	96.262	71.429	206	25	8	10	104
Touzan-hadaka 112	92.766	95.522	76.471	192	26	9	8	118
Steptoe	92.641	95.833	76.923	184	30	8	9	121
Minorimugi	92.444	96.277	72.973	181	27	7	10	128
Kashima Goal	92.405	94.175	80.645	194	25	12	6	116
Harumiyabi	92.369	97.748	48.148	217	13	5	14	104
Kanto-kawa 93	92.344	97.143	67.647	170	23	5	11	144
Kanto-kawa 96	92.276	96.602	70.000	199	28	7	12	107
Ichibanboshi	92.116	97.619	54.839	205	17	5	14	112
Haruhimeboshi	91.968	97.235	56.250	211	18	6	14	104
Kanto-kawa 97	91.892	95.699	72.222	178	26	8	10	131
Beaufiber	91.855	95.833	65.517	184	19	8	10	132
Suzukaze	91.837	94.86	70.968	203	22	11	9	108
Yumesakiboshi	91.827	95.402	73.529	166	25	8	9	145
Touzan-kawa 113	91.700	94.931	72.222	206	26	11	10	100
Nishinohoshi	91.525	96.602	56.667	199	17	7	13	117
Kanto-kawa 92	91.469	97.126	64.865	169	24	5	13	142
Silkysnow	91.286	96.517	65.000	194	26	7	14	112
Haruka Nijo	91.200	96.135	67.442	199	29	8	14	103
Sachiho Golden	91.111	95.833	63.636	184	21	8	12	128
Yokozuna	90.517	95.146	53.846	196	14	10	12	120
Shikoku-hadaka 84	90.393	96.447	53.125	190	17	7	15	124
Daishimochi	90.000	96.154	50.000	200	16	8	16	113
Haganemugi	72.959	75.882	53.846	129	14	41	12	156
Amagi Nijo	62.500	64.481	48.000	118	12	65	13	144
Asuka Golden	61.628	64.035	43.333	146	13	82	17	95
Tochinoibuki	58.203	62.441	37.209	133	16	80	27	97

## Discussion

### Availability of DNA Markers in Biparental Populations

In breeding programs, DNA markers are used to select polymorphisms associated with target traits, including a particular mutation of the gene or a genotype from a particular individual. A genome-wide distribution of markers and the marker density around a target gene are very important for these purposes. The aim of the present study was to estimate whether it is possible to achieve these marker conditions even among related strains, such as those used in Japanese barley breeding programs.

As Sato et al. (2011) reported, the availability of polymorphisms between closely related strains was limited in the prefixed SNP analysis of the Golden Gate assay, with just 386 of the 1488 SNPs showing a polymorphism between the cultivars Russia 6 and Mikamo Golden. In the RNA-Seq analysis performed here, we identified 5,102 polymorphisms between these strains (Supplementary Table 3), which were distributed throughout the genome (Supplementary Table 5). The level of polymorphism between Russia 6 and Mikamo Golden was lower than the average polymorphism between the strains investigated in the current study (range 156–26,075, average 11,140) (Supplementary Table 3); however, the availability of DNA markers was still sufficient for the selection of traits in breeding programs.

The range of pairwise polymorphisms identified using the RNA-Seq analysis indicates the efficiency of DNA marker generation, even between the related strains used in breeding programs; however, the relative number of polymorphisms was indeed lower within strains of same row type than between strains of the different row types. Polymorphisms are not likely to be abundant between identical haplotype regions of related strains. Although the positions of the transcribed regions were well distributed across the Morex genome sequence, we also identified gene-poor regions on the genome (e.g., on chromosome 4H). The low gene density around the centromeres meant that we could not assign transcripts to these regions; therefore, it is likely that our procedure for detecting polymorphisms using RNA-Seq could not generate markers for these gene-poor regions.

### Efficiency of the RNA-Seq Pipeline for the Generation of DNA Markers

Due to the limitations of the time required for calculations and the cost of sequencing multiple samples, we restricted the source of sequences to the transcripts generated in the RNA-Seq analysis. To improve the genotyping process, we indexed the reference genome using sambamba (Tarasov et al., 2015), which can index bam files in less time than samtools (Li, 2011). To save time when analyzing multiple samples, we used the transcribed regions from the reference genome sequence (Mascher et al., 2017). Our results showed that using only known HC loci did not fully cover the transcribed regions in our RNA-Seq sequences. The use of the transcripts and their ∼3-Kbp flanking regions reduced the size of the target sequences from 4.80 Gbp (whole genome) to just 0.59 Gbp, which had a major impact on the calculation time required for the mapping and genotyping processes. Our procedure using transcribed regions rather than the entire genome sequence halved the calculation time required for the mapping process and reduced the time required for genotyping by two thirds. Our comparison of the efficiencies of mapping the RNA-Seq data onto either the reference genome sequence or the transcribed regions did not change much in most samples in the meaning of mapping ratio.

### Quality and Application of RNA-Seq-Based DNA Markers in Breeding

We initially detected more than two million polymorphisms between Morex and the Japanese barley strains, which were distributed across the reference genome sequence. After the selection of polymorphisms with the thresholds of two or more reads and no neighboring polymorphisms around 60 bp, 226,702 polymorphisms were identified using 108 barley accessions. When we compared the genotypes of Morex and the Japanese barley strains, the number of polymorphisms in each pair were found to be relatively stable (ranging from 11,914 to 33,457); however, the RNA-Seq genotyping data did not include a large number of known polymorphisms. This was mainly due to the relatively low coverage of sequence reads (2.6–10.5 million read pairs) in this study, which was inevitable for an RNA-Seq analysis because of the low availability of reads from less highly expressed genes. As shown in Supplementary Figure 2, the plot of the read numbers and the numbers of SNPs among samples indicated that the numbers of SNPs were maintained larger at higher read number samples. The moderate level (ca. 100 markers per chromosome) of well distributed markers are ideal for trait mapping such as QTL (quantitative trait loci) analysis. More markers are needed for fine-mapping to candidate gene resolution and thus increasing the read depth would be advisable for the genome-wide genotyping. On the other hand, for the detection of core polymorphisms in a set of germplasms, such as strains used in breeding programs, it may be useful to focus more on the number of strains used than in the sequencing redundancy of a single strain, since common polymorphisms are likely present among the strains.

Several marker systems are currently available in barley. Illumina iSelect 50K array (Bayer et al., 2017) has SNPs with reference genome position but the polymorphisms are limited to the genotypes used to design the platform. GBS is a *de novo* detection of polymorphisms in genic and non-genic region with reference genome positions (Poland et al., 2012). Exome capture detects genomic sequence of the genic region (Mascher et al., 2013). Skim sequencing-based genotyping involves resequencing of multiple individuals followed by alignment of the reads to the reference sequence to genotype SNPs (Golicz et al., 2015). AmpliSeq is one of the best methods for the detection of targeted polymorphisms to date (Ogiso-Tanaka et al., 2019), and we estimated that there was an agreement rate of more than 90% in the core polymorphisms detected using RNA-Seq and AmpliSeq. As shown in Table 2, some of the accessions did not show strong agreement in genotyping results. We suspect that sources of RNA-Seq and Ampliseq were from different seed samples and their genotypes could be different. We also aggregated the accuracy by 387 Ampliseq target marker and found that 41 markers had missing data. These markers could be removed from the application. Of the 387 markers, 284 markers (82.1%) matched completely and other 34 markers showed less than five mismatches between RNA-Seq and AmpliSeq. Unlike a SNP array, AmpliSeq can detect not only a target SNP but also other flanking SNPs and indels within a target region. While the loci with multiple alleles represented 3,055 (1.3%) of the 226,702 total polymorphisms in RNA-Seq, relatively more of these sites were detected using AmpliSeq (30 out of 812; 3.7%). This difference might be caused by our avoidance of multiple-allelic sites in RNA-Seq in an attempt to retain reliable polymorphisms. AmpliSeq therefore identified more indel polymorphisms, which are generally less useful genomic markers than those based on SNPs, such as KASP, TaqMan and Fluidigm (Thomson et al., 2017). AmpliSeq requires information about the target polymorphisms before the analysis, and we therefore conclude that a possible DNA marker strategy for use in breeding programs would be to combine the detection of polymorphisms using RNA-Seq analysis and a subsequent marker detection using AmpliSeq.

## Author Contributions

KS and TT designed the experiments and wrote the manuscript. TY prepared the seed samples. KS performed the RNA-Seq. GI and EO-T performed the AmpliSeq.

## Conflict of Interest Statement

The authors declare that the research was conducted in the absence of any commercial or financial relationships that could be construed as a potential conflict of interest.

## Footnotes

1 https://doi.org/10.5447/IPK/2016/34

2 https://broadinstitute.github.io/picard/
