# Supplementary material for: Development of Genome-Wide SNP Markers for Barley via Reference- Based RNA-Seq Analysis
Source: Front Plant Sci. 2019 May 10;10:577. doi: 10.3389/fpls.2019.00577 (PMC6523396; doi:10.3389/fpls.2019.00577)
Supplement: Supplementary file 1 [file Presentation_1.PPTX]

## Slide 1
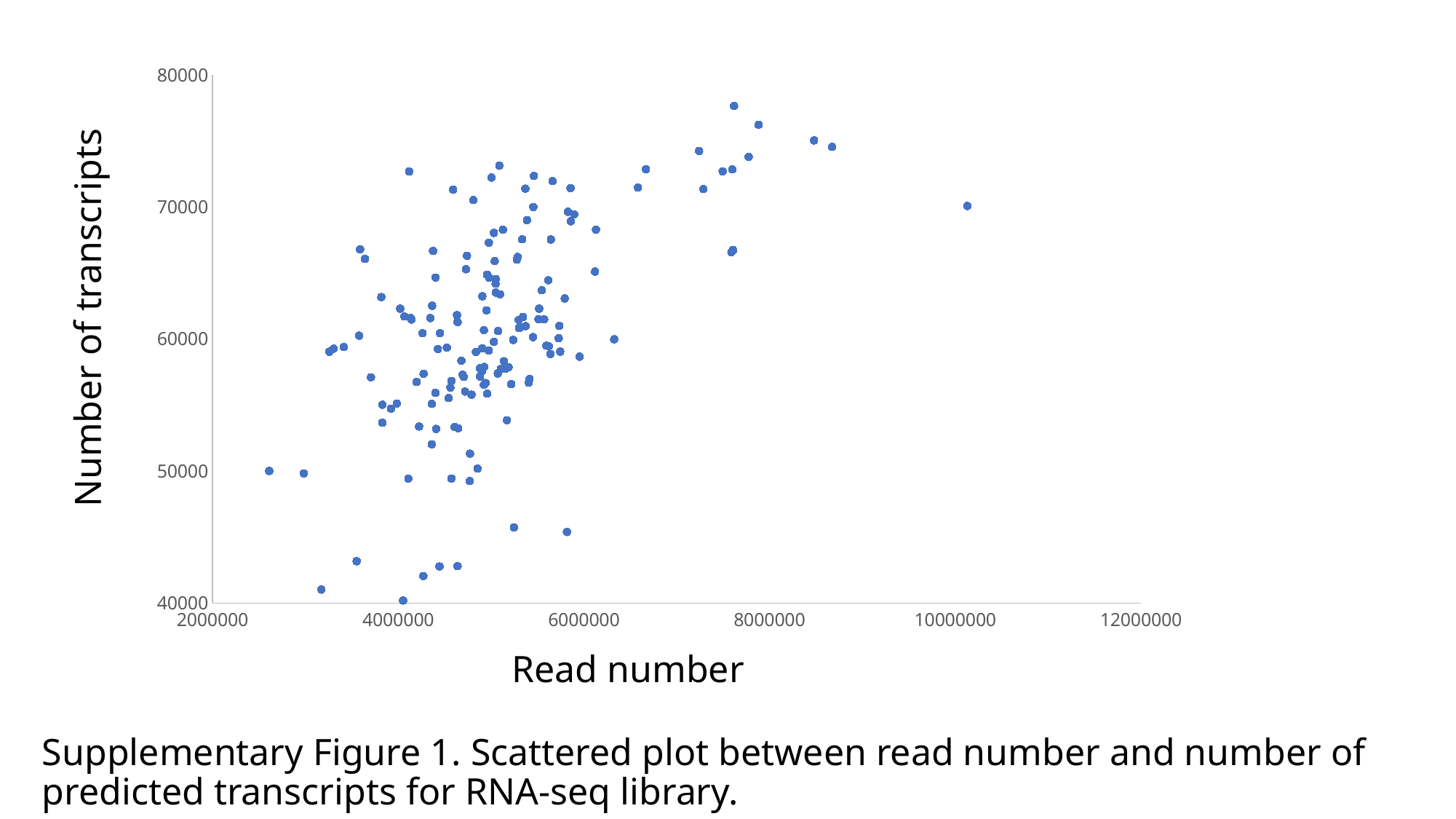

### Chart
| Category | |
|---|---|Number of transcripts
Read number
Supplementary Figure 1. Scattered plot between read number and number of predicted transcripts for RNA-seq library.

## Slide 2
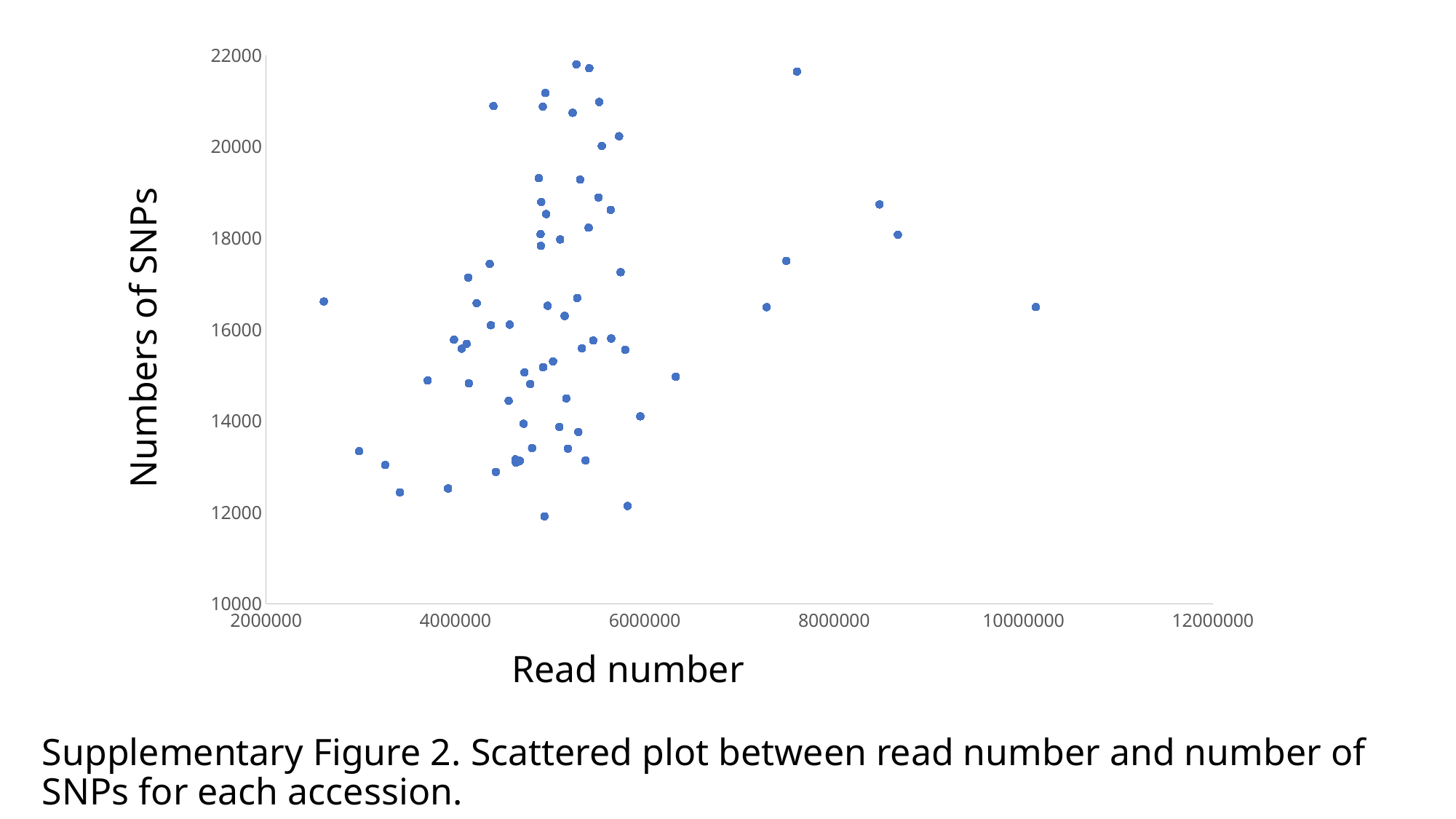

### Chart
| Category | |
|---|---|Numbers of SNPs
Read number
Supplementary Figure 2. Scattered plot between read number and number of SNPs for each accession.
