# Supplementary material for: Development of Genome-Wide SNP Markers for Barley via Reference- Based RNA-Seq Analysis
Source: Front Plant Sci. 2019 May 10;10:577. doi: 10.3389/fpls.2019.00577 (PMC6523396; doi:10.3389/fpls.2019.00577)
Supplement: Supplementary file 3 [file Table_2.DOCX]

Supplementary Table 2. The four step Perl scripts to filter genotyping results by > 1 read depth and no neighbor polymorphisms around 60 bp.

STEP1

#!/usr/bin/perl

$FILE = $ARGV[0];

open(OUT,">SNP.info_from$FILE");

open(IN,"$FILE");

while(<IN>){

chomp;

if(/^\#\#/){

}else{

print OUT "$_\n";

}

}

close(IN);

close(OUT);

STEP2

#!/usr/bin/perl

open(OUT,">SNP_nonredundant2.vcf");

open(IN,"Sample.list");

while(<IN>){

chomp;

@dat = split(" ",$_);

if(/(\S+)_L001/){

$sample = $1;

$id = "$dat[1]";

$group{$sample} = $id;

push(@$id,$sample);

$ID{$id} = "$dat[0]\_$dat[1]\_$dat[2]\_$dat[3]";

if($sude{$id} != 1){

push(@ID,$id);

$sude{$id} = 1;

}

}

}

close(IN);

open(INN,"SNP.info_fromgenotype2.vcf");

while(<INN>){

chomp;

@dat = split("\t",$_);

for($i = 0;$i <= 8;$i ++){

if($i == 0){

$ST = "$dat[$i]";

}else{

$ST .= "\t$dat[$i]";

}

}

if(/\#/){

foreach $ID(@ID){

print OUT "$ID\t";

}

print OUT "\n";

for($i = 9;$i <= 158;$i ++){

$N[$i] = $group{$dat[$i]};

}

}else{

$total ++;

undef(%G);

$OUTREF = 0;

$OUTSNP = 0;

$COUNT = 0;

for($i = 9;$i <= 158;$i ++){

$group = $N[$i];

if($dat[$i] =~ /0\/0\:(\d+)\,0/){

$RD = $1;

if($RD >= 2){

$genotype = "REF";

$OUTREF = 1;

$COUNT ++;

}else{

$genotype = "ND";

}

}elsif($dat[$i] =~ /1\/1\:0\,(\d+)/){

$RD = $1;

if($RD >= 2){

$genotype = "SNP";

$OUTSNP = 1;

$COUNT ++;

}else{

$genotype = "ND";

}

}elsif($dat[$i] =~ /\.\/\./){

$genotype = "";

}elsif($dat[$i] =~ /0\/0/ or $dat[$i] =~ /1\/1/){

$genotype = "ND";

}else{

$genotype = "HET";

}

if($G{$group} !~ /\w/){

$G{$group} = $genotype;

}elsif($G{$group} !~ /$genotype/){

$G{$group} = "DIF";

}

}

if($OUTREF == 1 and $OUTSNP == 1){

print OUT "$ST";

foreach $ID(@ID){

print OUT "\t$G{$ID}";

}

print OUT "\t$COUNT\n";

}else{

$DISCARD ++;

}

}

}

close(OUT);

close(INN);

STEP3

#!/usr/bin/perl

open(OUT,">SNP_genotype_nonredundant_2_6.vcf");

open(IN,"Sample.list");

while(<IN>){

chomp;

@dat = split("\t",$_);

if($dat[5] =~ /\?/){

}elsif($dat[5] =~ /6/){

$G{$dat[1]} = "6";

}elsif($dat[5] =~ /2/){

$G{$dat[1]} = "2";

}

}

close(IN);

open(INN,"SNP_nonredundant2.vcf");

while(<INN>){

chomp;

@dat = split("\t",$_);

if(/\#/){

print OUT "$_\t2REF\t2SNP\t2_MAF\t6REF\t6SNP\t6_MAF\n";

for($i = 9;$i <= 117;$i ++){

$G[$i] = $G{$dat[$i]};

}

}else{

$N{REF2} = 0;

$N{SNP2} = 0;

$N{REF6} = 0;

$N{SNP6} = 0;

for($i = 9;$i <= 117;$i ++){

if($dat[$i] =~ /REF/){

if($G[$i] =~ /6/){

$N{REF6} ++;

}elsif($G[$i] =~ /2/){

$N{REF2} ++;

}

}elsif($dat[$i] =~ /SNP/){

if($G[$i] =~ /6/){

$N{SNP6} ++;

}elsif($G[$i] =~ /2/){

$N{SNP2} ++;

}

}

}

$roku = $N{REF6} + $N{SNP6};

$ni = $N{REF2} + $N{SNP2};

if($roku > 0){

if($N{REF6} > $N{SNP6}){

$MAF6 = $N{SNP6} / $roku;

}else{

$MAF6 = $N{REF6} / $roku;

}

}else{

$MAF6 = "-";

}

if($ni > 0){

if($N{REF2} > $N{SNP2}){

$MAF2 = $N{SNP2} / $ni;

}else{

$MAF2 = $N{REF2} / $ni;

}

}else{

$MAF2 = "-";

}

print OUT "$_\t$N{REF2}\t$N{SNP2}\t$MAF2\t$N{REF6}\t$N{SNP6}\t$MAF6\n";

}

}

close(INN);

close(OUT);

STEP4

#!/usr/bin/perl

open(IN,"SNP.info_fromgenotype2.vcf");

open(OUT,">SNP_2_6_no60ALL.tab");

while(<IN>){

chomp;

@dat = split("\t",$_);

$id = $dat[0];

$$id[$dat[1]] = 1;

}

close(IN);

open(in,"SNP_genotype_nonredundant_2_6.vcf");

while(<in>){

$A = 0;

chomp;

@dat = split("\t",$_);

$id = $dat[0];

$pos = $dat[1];

$start = $pos - 60;

$l = $pos - 1;

if($start < 0){

$start = 1;

}

for($i = $start;$i <= $l;$i ++){

if($$id[$i] == 1){

$A ++;

}

}

$start = $pos + 1;

$l = $start + 60;

for($i = $start;$i <= $l;$i ++){

if($$id[$i] == 1){

$A ++;

}

}

if($A == 0){

print OUT "$_\n";

}

}

close(in);

close(OUT);
